# Utilizing Amari-Alpha Divergence to Stabilize the Training of Generative Adversarial Networks

**DOI:** 10.3390/e22040410

**Published:** 2020-04-04

**Authors:** Likun Cai, Yanjie Chen, Ning Cai, Wei Cheng, Hao Wang

**Affiliations:** 1 School of Information Science and Technology, ShanghaiTech University, Shanghai 201210, China; chenyj1@shanghaitech.edu.cn (Y.C.); ningcai@shanghaitech.edu.cn (N.C.); wanghao1@shanghaitech.edu.cn (H.W.); 2 Shanghai Institute of Microsystem and Information Technology, Chinese Academy of Sciences, Shanghai 200050, China; 3 University of Chinese Academy of Sciences, Beijing 100049, China; 4 NEC Laboratories America, Inc. (NEC Labs), NEC Corporation, Princeton, NJ 08540, USA; weicheng@nec-labs.com

**Keywords:** Alpha divergence, generative adversarial network, unsupervised image generation, deep neural networks

## Abstract

Generative Adversarial Nets (GANs) are one of the most popular architectures for image generation, which has achieved significant progress in generating high-resolution, diverse image samples. The normal GANs are supposed to minimize the Kullback–Leibler divergence between distributions of natural and generated images. In this paper, we propose the Alpha-divergence Generative Adversarial Net (Alpha-GAN) which adopts the alpha divergence as the minimization objective function of generators. The alpha divergence can be regarded as a generalization of the Kullback–Leibler divergence, Pearson $\chi^{2}$ divergence, Hellinger divergence, etc. Our Alpha-GAN employs the power function as the form of adversarial loss for the discriminator with two-order indexes. These hyper-parameters make our model more flexible to trade off between the generated and target distributions. We further give a theoretical analysis of how to select these hyper-parameters to balance the training stability and the quality of generated images. Extensive experiments of Alpha-GAN are performed on SVHN and CelebA datasets, and evaluation results show the stability of Alpha-GAN. The generated samples are also competitive compared with the state-of-the-art approaches.

## 1. Introduction

In recent years, deep learning has achieved incredible performance in theoretical research and many application scenarios, such as image classification [1,2], natural language processing [3], and speech recognition [4]. For high-dimensional data generation, deep neural networks-based generative models, particularly Generative Adversarial Networks (GANs) [5] have quickly become the most powerful model in unsupervised learning of image generation. Compared with the previous strategies, GANs have the power to generate high-resolution and vivid images. In a nutshell, GANs provide a framework to learn implicit distribution of a given target dataset X. There are typically two networks in GANs architecture: a generator network G(·) that produces vivid images, and a discriminator network D(·) that outputs scores on input images. The generator G(·) adopts a given latent noise *z* as input which is sampled from arbitrary distribution. The discriminator D(·) measures the difference between distributions of the generated fake samples and the real data. The core concept of GANs is to simultaneously train the discriminator and generator in turn by reducing the gap between two distributions under certain distance measurements. Recent work indicates that GANs have been making remarkable progress in a wide range of applications including image, video generation [6], image-to-image translation [7], and image super-resolution [8].

The original GAN model introduced by Goodfellow et al. proposed to minimize the Jensen–Shannon (JS) divergence between $p_{\mathrm{real}}$ and $p_{\mathrm{fake}}$, where $p_{\mathrm{real}}$ denotes the distribution of input real data and $p_{\mathrm{fake}}$ stands for the distribution of generated data. The cross-entropy loss is used for the output unit of discriminator. There exist two major challenges in the training of GANs. One is the performance balance between the generator and the discriminator. In the practical training process of GANs, the discriminators tend to learn better than the generators in most cases. As a result, it is difficult for the generators to study from data since the loss of discriminators becomes close to 0. Another problem is the model collapsing, which means the generated samples will collapse within a few data modes and become lack of diversity. In the literature, tremendous effort has been made to improve not only the ability to generate high-quality images but also convergence stability. For example, Least Square GAN (LSGAN) [9] leverages the Pearson $\chi^{2}$ divergence and adopts the least square loss for critic output. In [10], the authors proposed a new mechanism *f*-GAN to give an elegant generalization of GAN and extended the value function to arbitrary *f*-divergence. Compared with the original GANs, another contribution of *f*-GAN is that only single-step back-propagation is needed. Thus, there is no inner loop in the algorithm. However, the model collapsing problem remains unsolved by *f*-GAN [11].

To overcome the existing problems of GANs, one effective mechanism is proposed by Arjovsky et al.: the Wasserstein-GAN (i.e., WGAN) [12]. There are two main improvements in WGAN: a new objective based on the Wasserstein distance (or Earth Mover distance) and the weight clipping method. The Wasserstein distance has been proved to have better convergence performance than Kullback–Leibler divergence and Jensen–Shannon divergence in [12]. WGAN applies the approximated *Wasserstein distance* to estimate the distance between real and fake samples on a discriminator. The Kantorovich–Rubinstein duality is used to formulate the optimization. The original WGAN also requires the discriminator to be 1-Lipschitz continuous function, which can be achieved by clamping the model weights within a compact space ($W={\left[-0.01,0.01\right]}^{l}$), called weight clipping method. With these two methods to improve the model stability, WGAN can train the model till optimality to make the model less prone to collapse. However, this approach may lead to undesired behavior in practice [13]. To alleviate this effect, Gulrajani et al. proposed WGAN with gradient penalty (WGAN-GP) [13]. WGAN-GP introduces a soft penalty for the violation of 1-Lipschitz constraint, which guarantees high-quality image generation at the cost of the increasing computation complexity. Recently, many researchers have paid more attention to optimizing network architecture to improve training stability. For example, SN-GANs [14] create a novel weight normalization technique which is called Spectral Normalization to stabilize the training process. Although these approaches effectively improve the training stability, they provide less flexibility to strike a balance between the training stability and the desired quality of generated images.

In this paper, we propose Alpha Generative Adversarial Networks (Alpha-GANs) to train the generative model, which leverages the alpha divergence in information geometry [15]. We note that there is another so-called Alpha-GAN in [16]. However, there is no direct connection between these two models. Alpha-GAN in [16] is an application of GANs in natural image matting, while our proposed Alpha-GAN provides better objective function for the training scheme of GANs. Previous work has addressed the advances of alpha divergence and generalized it to many domains [17,18]. The alpha divergence can be seen as a generalization of multiple divergence functions, including Kullback–Leibler divergence [19], reverse KL divergence, Pearson $\chi^{2}$ divergence, and Hellinger distance. Each one corresponds to a unique value of alpha in the alpha-divergence family. We assume that a real-world data distribution is denoted by $p_{\mathrm{data}}$, the goal of generator network *G* is to recover $p_{\mathrm{real}}$ through its generated distribution $p_{\mathrm{fake}}$ such that $p_{\mathrm{fake}}$ is as close to $p_{\mathrm{real}}$ as possible. However, it is always a tricky problem to keep the balance between *G* and *D* in existing approaches. The key contribution of our method is to propose a new value function that generalizes the alpha divergence to a tractable optimization problem in the generative model. Our new formulation involves two-order hyper-parameters for $D(x_{\mathrm{real}})$ and $D(x_{\mathrm{fake}})$ respectively which control the trade-off between $p_{\mathrm{real}}$ and $p_{\mathrm{fake}}$ in the training process. Moreover, we provide theoretical analysis to suggest effective guidance to select these hyper-parameters to strike a balance between the training stability and the desired quality of generated images.

The main contributions of our work can be summarized as follows:

(1) We derive a new objective function for GANs inspired by the alpha divergence. Our new formulation preserves the order parameters of alpha divergence which can be further manipulated in the training progress. We note that *f*-GAN gives another generalization form of alpha divergence. Compared with the derivation in *f*-GAN, our Alpha-GAN has a more tractable formulation for optimization.
(2) The proposed adversarial loss function of Alpha-GAN has a similar formulation to Wasserstein-GAN. It introduces two hyper-parameters to strike a balance between *G* and *D*, and it can converge stably without any 1-Lipschitz constraints. Thus, Alpha-GAN can be regarded as an upgrade of WGAN, and the experimental results show the advanced performance of our model.
(3) Through our new value function, we dig out a trade-off between training stability and quality of generated images in GANs. The two properties can be directly controlled by adjusting the hyper-parameters in the objective.

The rest of this paper is organized as follows. Section 2 briefly reviews the background of alpha divergence and some state-of-the-art architectures of GANs. More details about our proposed Alpha-GAN are formally stated in Section 3. In Section 4, experimental results are shown. Finally, we conclude our work in Section 5.

## 2. Background and Related Work

In this section, we introduce necessary background information regarding the alpha-divergence family and entropy and explain its relationship with novel generative models. Then, we go over some state-of-the-art GANs in the literature.

### 2.1. Entropy and Alpha Divergence

Before introducing the alpha divergence, we first review the concept of information entropy. The information entropy is proposed by Shannon, which is an important definition in information theory. Give a random variable *X* and its probability density p(x), the entropy can be defined as:

(1) $$H\left(X\right)=\int_{x}p\left(x\right)I\left(x\right)=-\int_{x}p\left(x\right)\log p\left(x\right)dx$$

It can be seen that if p(x) gets closer to uniform distribution, the corresponding entropy will be greater. Although information entropy has few direct applications in machine learning, the cross-entropy is widely used in machine learning which is a derived from basic entropy:

(2) $$H_{\mathrm{CE}}\left(p,q\right)=E_{p}\left[-\log q\right]=-\int_{x}p\left(x\right)\log q\left(x\right)dx$$

Cross-entropy is used to evaluate the difference between two distributions.

Kullback–Leibler (KL) divergence is another method to measure the disparity of distributions, which is also called relative entropy. KL divergence is generalized as the value function of original GANs. Given two probability densities *p* and *q* of random variable *X*, the KL divergence can be defined as:

(3) $$D_{\mathrm{KL}}\left(p∥q\right)=-\int_{x}p\left(x\right)\log\frac{q(x)}{p(x)}dx=\int_{x}p\left(x\right)\log\frac{p(x)}{q(x)}dx$$

We can find that there is a relationship between entropy, cross-entropy and KL divergence:

(4) $$H_{\mathrm{CE}}\left(p,q\right)=H\left(p\right)+D_{\mathrm{KL}}\left(p∥q\right)$$

Divergence function is a critical part of the overall framework of the GANs, since it is used to measure the difference between two data distributions $p_{\mathrm{real}}$ and $p_{\mathrm{fake}}$. The regular GANs use Kullback–Leibler divergence as the critic measurement, which proves not to be the optimal choice in previous studies. In this work, we use the alpha divergence, and derive a new objective for GANs in Equation (13). We first give a brief review of alpha divergence upon which our Alpha-GAN model is based. Here, we mainly introduce two kinds of alpha divergence: Amari-alpha divergence [15] and Rényi-alpha divergence [20]. Considering two probability densities *p* and *q* of random variable θ, these two forms of alpha divergence can be defined on {α:α∈R\{0,1}} as follows:

- Amari-alpha divergence:
  (5) $$D_{A}\left[p∥q\right]=\frac{1}{\alpha (\alpha -1)}\left(\int p{\left(\theta \right)}^{\alpha }q{\left(\theta \right)}^{1-\alpha }d\theta -1\right).$$
- Rényi-alpha divergence:
  (6) $$D_{R}\left[p∥q\right]=\frac{1}{\alpha -1}\log\left(\int p{\left(\theta \right)}^{\alpha }q{\left(\theta \right)}^{1-\alpha }d\theta \right).$$

These divergences are related to the Chernoff α-coefficient $c_{\alpha }\left(p:q\right)=\int p{\left(\theta \right)}^{\alpha }q{\left(\theta \right)}^{1-\alpha }d\theta$ [21]. Please note that when α→0, the Kullback–Leibler divergence can be recovered from both Amari and Rényi divergence while α→1 leads to the reverse Kullback–Leibler divergence [22,23]. We present some other special cases of the Amari-alpha-divergence family in Table 1. It can be regarded as the final criterion of our Alpha-GAN with some simple manipulations. We also include some useful properties of Amari-alpha divergence [23] in the following:

**Theorem** **1** (Convexity)**.** *Given two distributions p and q, the alpha divergence $D_{A}\left[p∥q\right]$ is a convex function with respect to both p and q. So for any 0≥λ≥1, we have*

$$D_{A}\left[\lambda p_{1}+\left(1-\lambda \right)p_{2}∥\lambda q_{1}+\left(1-\lambda \right)q_{2}\right]\leq \lambda D_{A}\left[p_{1}∥q_{1}\right]+\left(1-\lambda \right)D_{A}\left[p_{2}∥q_{2}\right].$$

**Theorem** **2** (Strict Positivity)**.** *The alpha divergence is a strictly positive function $D_{A}\left[p∥q\right]\geq 0$, and it has a unique minimum $D_{A}\left[p∥q\right]=0$ if and only if p=q.*

**Theorem** **3** (Duality)**.** *$D_{A}^{\left(\alpha \right)}\left[p∥q\right]=D_{A}^{\left(1-\alpha \right)}\left[q∥p\right]$.*

We will show how to adopt Amari-alpha divergence in our proposed GAN objective in Section 3. An effective guidance to select appropriate hyper-parameters for the rich alpha-divergence family is also provided.

### 2.2. Generative Adversarial Networks

**Figure 1:**
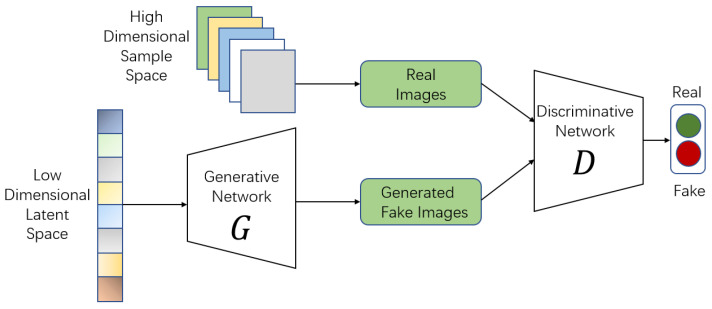
The architecture of vanilla GANs.

In recent years, generative adversarial networks (GANs) have been one of the most attractive architectures in machine-learning systems. Since it was proposed by Goodfellow et al. in 2014 [5], tremendous variants of GAN have been produced by researchers. Most of them preserve the initial framework of vanilla GAN, which consists of two neural networks: a generator *G* and a discriminator *D*. *G* and *D* will adversarially learn from each other during the training phase. Figure 1 illustrates the schematic diagram of vanilla GANs, where *G* generates a fake image from a random latent code $z∼p_{z}$ and *D* learns to distinguish between real and fake samples. The key idea of GANs is usually defined as a game-play problem with a min–max objective. Researchers aim to obtain the optimal generator, which can generate high-resolution vivid images similar to natural images by fine-tuning the hyper-parameters properly. Next, we briefly show some popular objectives used to train a generative model.

#### 2.2.1. Vanilla GAN

The original GAN proposed in [5] can be defined as a contest between two networks *G* and *D*. The min–max objective is formally defined as follows:

(7) $$\min_{G}\max_{D}E_{x∼p_{\mathrm{real}}}\left[\log(D(x))\right]+E_{x∼p_{G}}\left[\log(1-D(x))\right],$$

where x stands for the input image, $p_{\mathrm{real}}$ and $p_{\mathrm{fake}}$ represent the distribution of real-world and the generated data respectively. This objective follows the formulation of binary cross-entropy loss. The outputs of discriminator D(·) are confined within [0,1] through a sigmoid activation unit. The final critic value function of vanilla GANs can be formulated as the Jensen–Shannon divergence between $p_{\mathrm{real}}$ and $p_{\mathrm{fake}}$:

(8) $$\begin{array}{cc} C(G) & =E_{x∼p_{\mathrm{real}}}\left[\log\frac{p_{\mathrm{real}}\left(x\right)}{p_{\mathrm{real}}\left(x\right)+p_{\mathrm{fake}}\left(x\right)}\right]+E_{x∼p_{\mathrm{fake}}}\left[\log\frac{p_{\mathrm{fake}}\left(x\right)}{p_{\mathrm{real}}\left(x\right)+p_{\mathrm{fake}}\left(x\right)}\right]\\ \; & =2\cdot JS(p_{\mathrm{real}}∥p_{\mathrm{fake}})-\log4, \end{array}$$

The above min–max optimization problem is a popular mechanism in deep generative models. However, this model suffers from the unbalanced training problem of two neural networks.

#### 2.2.2. LSGAN

One of the GANs’ variants is LSGAN [9]. Compared with vanilla GANs, LSGANs substitute the binary cross-entropy loss with a least square loss, which has better properties for optimization and is less likely to saturate. LSGAN adopts the Pearson $\chi^{2}$ divergence as the decision criterion. It is computed as follows:

(9) $$\begin{array}{cc} \min_{D}V_{\mathrm{LSGAN}}\left(D\right) & =\frac{1}{2}E_{x∼p_{\mathrm{real}}}\left[{\left(D\left(x\right)-1\right)}^{2}\right]+\frac{1}{2}E_{z∼p_{z}}\left[{\left(D\left(G\left(z\right)\right)\right)}^{2}\right],\\ \min_{G}V_{\mathrm{LSGAN}}\left(G\right) & =\frac{1}{2}E_{z∼p_{z}}\left[{\left(D\left(G\left(z\right)\right)-1\right)}^{2}\right], \end{array}$$

where $z∼p_{z}$ is the input latent noise of generator. LSGANs adopt the Pearson $\chi^{2}$ divergence as the decision criterion:

(10) $$\begin{array}{cc} C(G) & =\frac{1}{2}\int_{X}\frac{(2p_{\mathrm{fake}}\left(x\right)-{\left(p_{\mathrm{real}}\left(x\right)+p_{\mathrm{fake}}\left(x\right)\right)}^{2}}{p_{\mathrm{real}}\left(x\right)+p_{\mathrm{fake}}\left(x\right)}dx\\ \; & =\frac{1}{2}\chi_{\mathrm{Pearson}}^{2}\left(p_{\mathrm{real}}+p_{\mathrm{fake}}∥2p_{\mathrm{fake}}\right), \end{array}$$

Nowozin et al. proposed a new mechanism *f*-GAN to give an elegant generalization of GAN and extended the value function to arbitrary *f*-divergence including $\chi^{2}$ divergence in [10]. However, there still exists model collapsing for LSGANs and *f*-GAN.

#### 2.2.3. Wasserstein-GAN

To further enhance the stability of GANs, Arjovsky et al. applied the Earth Mover (also called Wasserstein-1) distance, which is used to measure optimal transport cost between two distributions [12]. The Wasserstein distance is defined as:

(11) $$W\left(P_{r},P_{g}\right)=\underset{\gamma \in \Pi (P_{r},P_{g})}{\inf}E_{(x,y)∼\gamma }\left[∥x-y∥\right].$$

where $\Pi (P_{r},P_{g})$ represents all joint distributions of $P_{r}$ and $P_{g}$. Wasserstein GANs also employ the Kantorovich–Rubinstein duality of Wasserstein-1 distance to construct the value function:

(12) $$\min_{G}\max_{D}E_{x∼P_{r}}\left[D(x)\right]-E_{x∼P_{G}}\left[D(x)\right].$$

where *D* should be a 1-Lipschitz function and the weight parameters are clipped within the numerical interval [−c,c]. Gulrajani et al. proposed WGAN with gradient penalty (WGAN-GP) [13]. WGAN-GP introduces a soft penalty for the violation of 1-Lipschitz constraint, which guarantees high-quality image generation at the cost of the increasing computation complexity. SN-GANs [14] create a novel weight normalization technique which is called Spectral Normalization to stabilize the training process. Although these approaches effectively improve the training stability, they provide less flexibility to strike a balance between the training stability and the desired quality of generated images.

## 3. Proposed Method

We introduce our Alpha-GAN, a novel architecture of generative model based upon the minimization of alpha divergence [15]. The exact formulation of Alpha-GAN is defined in Equation (13) and we will show the relationship between Alpha-GAN and alpha divergence in Section 3.2.

### 3.1. Alpha-GAN Formulation

Inspired by the alpha divergence, we propose our new framework: the Alpha-GAN. In contrast to original GANs, Alpha-GAN removes the sigmoid output layer in discriminator network and substitutes the binary cross-entropy loss with our power function formulation. The proposed method further introduces two more hyper-parameters compared to WGAN. Specifically, Alpha-GAN model solves the following optimization problem:

(13) $$\begin{array}{c} \min_{G}\max_{D}V_{\mathrm{Alpha}-\mathrm{GAN}}\left(D,G\right)=E_{x∼p_{\mathrm{real}}\left(x\right)}\left[{\left|D\left(x\right)\right|}^{a}\right]-E_{z∼p_{z}}\left[{\left|D\left(G\left(z\right)\right)\right|}^{b}\right]. \end{array}$$

Please note that a,b are two-order indices for D(x) and D(G(z)) respectively. They are hyper-parameters introduced to balance the emphasis on D(x) and D(G(z)) during training process. To enhance the convergence stability, our proposed method only considers a,b>0 in order to avoid the case that a term like $\frac{1}{D^{a}}$ appears in the loss function when a≤0 or b≤0. When the discriminator’s output is smaller than 1, the loss value would be extremely large and accordingly the model would become less stable and hard to converge in training phase. Another update is that we take the absolute value of the discriminator output. Otherwise, the output would produce a trivial solution when a<1 or b<1. It seems like the objective function of Alpha-GAN is not immediately related to the formulation of alpha divergence in Equation (5). We will give the detailed theoretical analysis of how to derive Alpha-GAN from alpha divergence in Section 3.2. The training scheme of our Alpha-GAN is shown in Algorithm 1.

In [10], *f*-GAN also provides a value function related to alpha divergence. The authors generalize *f*-divergence to GAN objectives via a variational lower bound. The *f*-GAN objective with respect to alpha divergence can be defined as:

(14) $$\begin{array}{cc} V_{f-\mathrm{GAN}} & =E_{x∼p_{\mathrm{real}}}\left[g_{f}\left(V\left(x\right)\right)\right]+E_{x∼p_{\mathrm{fake}}}\left[-f^{*}\left(g_{f}\left(V\left(x\right)\right)\right)\right],\\ f^{*}\left(t\right) & =\frac{1}{\alpha }{\left(t\left(\alpha -1\right)+1\right)}^{\frac{\alpha }{\alpha -1}}-\frac{1}{\alpha } \end{array}$$

where V(x) denotes the output of last layer of discriminator network and $g_{f}$ is the output activation. For different values of α in alpha divergence, the activation $g_{f}$ also has different formulations:

(15) $$\begin{array}{cc} g_{f}\left(v\right)=\frac{1}{1-\alpha }-\log\left(1+\exp\left(-v\right)\right),\; & \mathrm{for}\;\alpha <1,\alpha \neq 0\\ g_{f}\left(v\right)=v,\; & \mathrm{for}\;\alpha >1 \end{array}$$

The above objective has complex formulations and constraints, which makes it inconvenient for optimization in deep generative models. In addition, the severe model collapsing problem remains unsolved. In our proposed method, a simplified objective function is given with a similar induction process as the vanilla GANs, which has a more elegant form to balance output between stability and quality. A detailed analysis of the derivation will be shown in the next section.
**Algorithm 1** Training scheme of Alpha-GAN.**Input**: Batch size *m*, target distribution $p_{\mathrm{real}}$, latent noise distribution $p_{z}$, input noise *z*, Adam optimizer with $\alpha ,\beta_{1}=0.5,\beta_{2}=0.999$, hyper-parameters a,b, discriminator network $D_{\phi }$ and generator network $G_{\theta }$, absolute function abs(·).**Output**: Optimal generator $G_{\theta }$.1:**while** Training scheme of Alpha-GAN **do**2: Sample $x_{i}^{r}∼p_{\mathrm{real}},i=1,\cdots ,m.$3: Sample $z_{i}∼p_{z},i=1,\cdots ,m.$4: $x_{i}^{g}\leftarrow G_{\theta }\left(z_{i}\right),i=1,\cdots ,m$5: $\phi \leftarrow \mathrm{Adam}(-\frac{1}{m}\sum_{i=1}^{m}\nabla_{\phi }\left[\mathrm{abs}{\left(D_{\phi }\left(x_{i}^{r}\right)\right)}^{a}\right])$6: $\phi \leftarrow \mathrm{Adam}(\frac{1}{m}\sum_{i=1}^{m}\nabla_{\phi }\left[\mathrm{abs}{\left(D_{\phi }\left(x_{i}^{g}\right)\right)}^{b}\right])$7: $\theta \leftarrow \mathrm{Adam}(\frac{1}{m}\sum_{i=1}^{m}\nabla_{\theta }\left[\mathrm{abs}{\left(D\left(x_{i}^{g}\right)\right)}^{b}\right])$8:**end while**9:**return**$G_{\theta }$

### 3.2. Theoretical Analysis

The original GAN model from Ian Goodfellow et al. proposed to minimize the Jensen–Shannon divergence:

(16) $$C\left(G\right)=2\cdot JS\left(p_{\mathrm{real}}∥p_{\mathrm{fake}}\right)-\log4.$$

The JS divergence can be written as the summation of KL divergence. Therefore, the final criterion of original GAN is the KL distance between the distributions of the ground-truth image and the generated one. However, there are many research results ([12]) showing that KL divergence is not a good objective for optimization. The alpha divergence employed in our approach can be seen as a generalization of KL divergence and we have already presented some basic properties of the alpha divergence in Section 2.1.

Next, we show how Alpha-GAN is related to the alpha divergence mentioned in Equation (5). We first give the proof of optimal discriminator $D^{*}$ for arbitrary generator *G*.

**Theorem** **4.** For any fixed generator G and a<b, we prove the optimal discriminator $D^{*}$ as:

(17) $$D^{*}\left(x\right)={\left(\frac{b\cdot p_{\mathrm{fake}}\left(x\right)}{a\cdot p_{\mathrm{real}}\left(x\right)}\right)}^{\frac{1}{a-b}}.$$

**Proof.**  To prove the optimal $D^{*}$ defined in Equation (17), we show that the objective function for discriminator *D* is to maximize the following Equation:

(18) $$\begin{array}{cc} V(D,G) & =\int_{x}p_{\mathrm{real}}{\left(x\right)|D\left(x\right)|}^{a}dx-\int_{z}p_{z}{\left|D\left(G\left(z\right)\right)\right|}^{b}dz\\ \; & =\int_{x}p_{\mathrm{real}}{\left(x\right)|D\left(x\right)|}^{a}-p_{\mathrm{fake}}\left(x\right){\left|D\left(x\right)\right|}^{b}dx. \end{array}$$

In Section 3.1, we already stated that we only consider a,b>0 and we keep this setting in the proof. Then for a<b, the upper function is concave in [0,∞). We can take a derivative of it with respect to *D* and the optimal $D^{*}$ in Equation (17) will be obtained. Since the optimal solution only lies within [0,∞), we take the absolute value of critic output. □

After that, we substitute the optimal $D^{*}\left(x\right)$ into the initial objective function as defined in Equation (13). We can reformulate it as follows:

(19) $$\begin{array}{cc} C(G) & =\max_{D}V\left(D,G\right)\\ \; & =E_{x∼p_{\mathrm{real}}}\left[D_{G}^{*}{\left(x\right)}^{a}\right]-E_{z∼p_{z}}\left[D_{G}^{*}{\left(G\left(z\right)\right)}^{b}\right]\\ \; & =E_{x∼p_{\mathrm{real}}}\left[D_{G}^{*}{\left(x\right)}^{a}\right]-E_{x∼p_{\mathrm{fake}}}\left[D_{G}^{*}{\left(x\right)}^{b}\right]\\ \; & =\int_{x}p_{\mathrm{real}}\left(x\right){\left(\frac{b\cdot p_{\mathrm{fake}}\left(x\right)}{a\cdot p_{\mathrm{real}}\left(x\right)}\right)}^{\frac{a}{a-b}}dx-\int_{x}p_{\mathrm{fake}}\left(x\right){\left(\frac{b\cdot p_{\mathrm{fake}}\left(x\right)}{a\cdot p_{\mathrm{real}}\left(x\right)}\right)}^{\frac{b}{a-b}}dx\\ \; & =\int_{x}{\left(\frac{b}{a}\right)}^{\frac{a}{a-b}}p_{\mathrm{real}}{\left(x\right)}^{\frac{b}{b-a}}p_{\mathrm{fake}}{\left(x\right)}^{\frac{a}{a-b}}dx-\int_{x}{\left(\frac{b}{a}\right)}^{\frac{b}{a-b}}p_{\mathrm{real}}{\left(x\right)}^{\frac{b}{b-a}}p_{\mathrm{fake}}{\left(x\right)}^{\frac{a}{a-b}}dx. \end{array}$$

If we denote $\alpha =\frac{b}{b-a}$, $1-\alpha =\frac{a}{a-b}$, and set $c={\left(\frac{b}{a}\right)}^{\frac{a}{a-b}}-{\left(\frac{b}{a}\right)}^{\frac{b}{a-b}}$, we can obtain from above Equation:

(20) $$\begin{array}{cc} C(G) & =c\cdot \int_{x}p_{\mathrm{real}}{\left(x\right)}^{\alpha }\cdot p_{\mathrm{fake}}{\left(x\right)}^{1-\alpha }dx\\ \; & =c\cdot (\alpha \left(\alpha -1\right)D_{A}\left[p_{\mathrm{real}}∥p_{\mathrm{fake}}\right]+1). \end{array}$$

The final training criterion of generator *G* shown above can be seen as a linear transformation of the alpha divergence. Hence, our Alpha-GAN aims to reduce the distance measured by alpha divergence. We can manipulate the order α of divergence function through adjusting the value of hyper-parameters *a* and *b*.

### 3.3. Selection of Hyper-Parameters

Our Alpha-GAN uses two hyper-parameters *a* and *b* to control the update rate of D(x) and D(G(z)). The derivation in Equations (19) and (20) already states that changing a,b is equivalent to adjusting the order of alpha divergence. The relationship between *a* and *b* represents the preferences of model on real images or fake images. In practice, it is flexible for users to balance the training stability and the desired quality of generated images according to their specific requirements. One key problem is how to select proper hyper-parameters to obtain the optimal model. Here we give some useful suggestions on parameters selection:

- $\frac{b}{2}\leq a\leq b$: To prove the optimal discriminator $D^{*}$ in Theorem 4, the hyper-parameters have been set to a<b to satisfy the optimal condition. In the experiments of Alpha-GAN, we find that the scope can be reduced to $\frac{b}{2}\leq a\leq b$. This will help us to determine ratio between two parameters in the applications. Noting that a=b could also lead to good generation results while it does not satisfy the optimal condition in Theorem 4. We interpret this phenomenon as that the Alpha-GAN has a similar formulation like WGAN when a=b. These can be written as:
  (21) $$\begin{array}{cc} V_{\mathrm{Alpha-GAN}}\left(D,G\right) & =E_{x∼p_{\mathrm{real}}}\left[|D(x)|\right]-E_{z∼p_{z}}\left[|D(G(z))|\right],\\ V_{\mathrm{WGAN}}\left(D,G\right) & =E_{x∼p_{\mathrm{real}}}\left[D(x)\right]-E_{z∼p_{z}}\left[D(G(z))\right] \end{array}$$
  We believe that the setting of a=b will have some similar convergence properties like WGAN.
- a,b≥0.4: For the training stability of Alpha-GAN model, we only consider a,b>0 to avoid forms like $\frac{1}{D^{a}}$ as stated in Section 3.1. Otherwise, the loss will be extremely unstable when D≪0. In evaluation experiments, when we set a,b<0.4, the model cannot converge successfully, and the generated images are very blurred. The small values of parameters mean that the gradients feedback will be multiplied by a small coefficient in back-propagation. It is hard for the generator and discriminator to learn useful information from image data in such settings. Thus, we recommend setting the parameters to a,b≥0.4.
- a,b≤1: This suggestion is also summarized from the experimental results, and may not always be valid. In the image generation experiments of Alpha-GAN, we find that the loss curves fluctuate largely when a,b>1. However, the quality of generated images is not too bad. We believe the model will be difficult to converge well when it is faced with more complex problems, such as larger image datasets.

One way to select proper hyper-parameters is referring to the special cases of alpha divergence as shown in Table 1. For example, we observe that a good convergence result will be obtained when the parameters are set as 2a=b, which corresponds to the Pearson $\chi^{2}$ divergence in alpha-divergence family. We already denote $\alpha =\frac{b}{b-a}$ in Equation (20). Then for 2a=b, we can get:

(22) $$\begin{array}{cc} C(G) & =c\cdot \int_{x}p_{\mathrm{real}}{\left(x\right)}^{2}\cdot p_{\mathrm{fake}}{\left(x\right)}^{-1}dx\\ \; & =c\cdot (D_{\chi^{2}}\left[p_{\mathrm{real}}∥p_{\mathrm{fake}}\right]+1). \end{array}$$

And we also suggest a,b≤1 in the previous analysis. Here we further simplify the parameters as $a=\frac{1}{2},b=1$. Then, the adversarial loss of Alpha-GAN can be written as:

(23) $$E_{x∼p_{\mathrm{real}}\left(x\right)}\left[D{\left(x\right)}^{\frac{1}{2}}\right]-E_{z∼p_{z}}\left[D(G(z))\right].$$

Noting that we do not claim the values of $a=\frac{1}{2},b=1$ or Pearson $\chi^{2}$ divergence are optimal for Alpha-GAN. It is one of our observations that such parameter settings can bring stable convergence performance in applications, thus we give a piece of reasonable advice to initialize *a* and *b*. In [9], the author employs Pearson $\chi^{2}$ divergence to generalize the LSGAN. Compared with the adversarial loss of LSGAN in Equation (9), our model has a totally different formulation in Equation (23). Our Alpha-GAN is derived from a special case of alpha divergence, not directly from $\chi^{2}$ divergence. We also evaluate the effects of different settings under diverse hyper-parameters and the effectiveness of our mechanism will be shown in Section 4.

## 4. Experiments

In this section, we conduct extensive experiments to evaluate the proposed method. We compare Alpha-GAN with some baseline models to show the competitive results of our approach. The algorithms are all implemented with PyTorch [24] in this section. The source code can be found in https://github.com/cailk/AlphaGAN.

### 4.1. Datasets

There are three datasets involved in our paper, including the handwritten digital dataset MNIST [25], and two real-world image datasets SVHN [26] and CelebA [27].

- **MNIST**: MNIST is a widely used database of handwritten digits, which contains a training set of 60,000 images and a test set of 10,000 images. There are 10 labels from ‘0’ to ‘9’ for dataset and all digits are normalized to 28×28. We use MNIST to evaluate the trade-off effect between two hyper-parameters in the value function.
- **SVHN**: SVHN is a real-world color image dataset obtained from house numbers in Google Street View images. Its training set contains 73,257 32×32 digital images. SVHN dataset is similar to MNIST, but comes from a harder problem since all digits are sampled in natural scene.
- **CelebA**: Last dataset used in this paper is CelebA, which is a large-scale face attribute dataset with more than 200,000 images. Samples are all 64×64 color celebrity images. CelebA is an important dataset in the scenario of image generation since it only contains information of face attribute and is easy to learn for GANs.

### 4.2. Model Architectures and Implementation Details

The architecture of our generator and discriminator is designed based on the InfoGAN [28]. The generator network is fed by a latent variable $z∼N_{128}\left(0,I\right)$. It contains a fully connection layer that upscales the input tensor to size 512×2×2, four transposed convolution layers (kernel size = 4×4, stride = 2, padding = 1) and the tanh activation layer. The discriminator network consists of 4 convolution layers that extract features from 32×32 inputs. ReLU activation function is used after each layer in generator network and Leaky-ReLU for discriminator network. Batch normalization is employed in each layer of both networks.

For our Alpha-GAN in Equation (13), we remove the last activation layer of discriminator like WGAN [12], and we apply an abs function to the critic output. We employ Adam optimizer [29] with learning rate of 0.0002 and decay rates of $\beta_{1}=0.5,\beta_{2}=0.999$ to train the generator network. In addition, the discriminator network is also trained using an Adam optimizer with learning rate of 0.0002. The total number of epochs is 50 for MNIST, SVHN, and 30 for CelebA. All experiments are conducted in a machine with one NVIDIA GTX 1080 GPU.

### 4.3. Evaluation Metrics

Measuring the quality of generated images is usually a more tricky and challenging problem than simply generating vivid images. It is almost impossible to directly establish an objective on the space of natural and generated images. To measure the quality of generated image samples, we employ the Fréchet Inception Distance (FID) proposed in [30], which is a commonly used metric for GANs. The FID is supposed to be more advanced than Inception Score (IS) [31] which is another metric to evaluate deep generative models. Suppose two multivariate Gaussians $X_{\mathrm{real}}∼N\left(\mu_{1},\Sigma_{1}\right)$ and $X_{\mathrm{fake}}∼N\left(\mu_{2},\Sigma_{2}\right)$ are the 2048-dimensional activation outputs of the Inception-v3 [32] pool_3 layer for real and generated samples respectively. The FID can be defined as follows:

(24) $$\mathrm{FID}=∥\mu_{1}-\mu_{2}{∥}^{2}+\mathrm{Tr}\left(\Sigma_{1}+\Sigma_{2}-2{\left(\Sigma_{1}\Sigma_{2}\right)}^{\frac{1}{2}}\right).$$

FID compares the statistics of fake images to real ones, instead of only evaluating the generated samples. Thus, FID will give a more reliable standard to measure the effect of GANs. For FID score, low is better, meaning real and generated samples are more similar, measured by the distance between their activation distributions.

### 4.4. The Influence of Hyper-Parameters

In our Alpha-GAN, we introduce two hyper-parameters a,b to the objective function, and we interpret them as how favorite the model want to learn from real and fake data distribution. To verify the influence of changing values of *a* and *b* in Equation (13), we conducted extensive experiments on MNIST dataset to demonstrate the trade-off between $p_{\mathrm{real}}$ and $p_{\mathrm{fake}}$.

**Figure 2:**
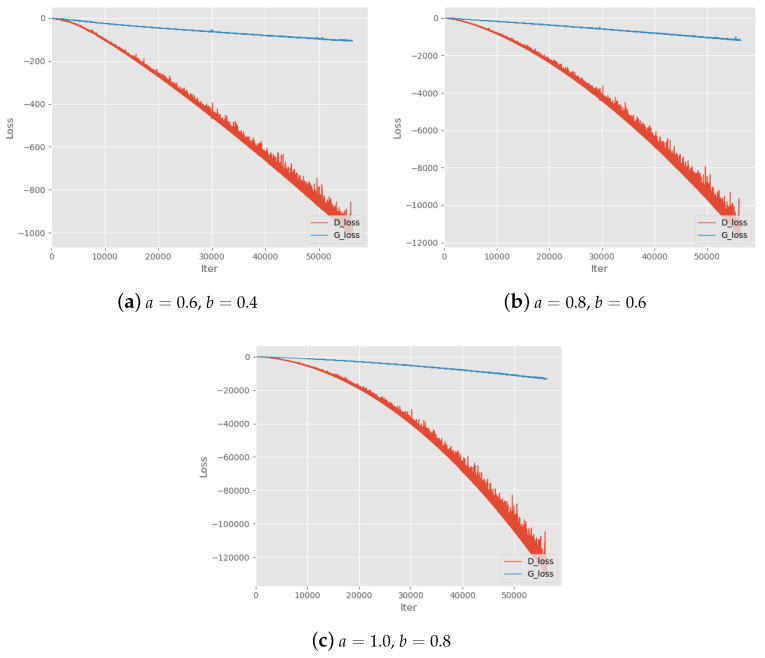
Training Loss curves with parameters settings a>b. a,b denote the two hyper-parameters in the objective function.

**Figure 3:**
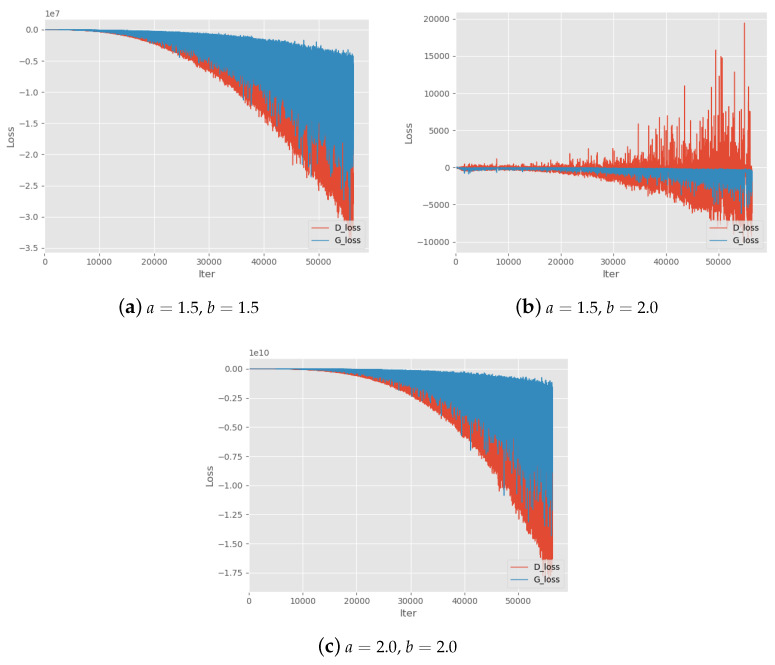
Training Loss curves with parameters settings a,b>1. a,b denote the two hyper-parameters in the objective function.

First, we test various parameter settings of Alpha-GAN to evaluate the basic convergence performance on different values of parameters *a* and *b*. The results can be found in Table 2. *a* and *b* are the two parameters in adversarial loss of Alpha-GAN. The symbol ‘*√*’ denotes the models with corresponding parameter setting can converge normally and generate high-quality digital images. ’–’ means the quality of generated by corresponding models is slightly poor. In addition, ‘×’ means the model cannot converge and generated samples are blurred. We also find that when a<0.4 or b<0.4, the model will not converge. Thus, we recommend keeping the parameters greater than or equal to 0.4. In the theoretical analysis of Alpha-GAN, we suppose a<b to ensure the concavity of objective function. We can see that almost all settings on a>b will lead to poor model performance except for (0.6,0.4),(0.8,0.6),(1.0,0.8). Figure 2 shows the training loss curves of these settings, which means that models do not converge during the training progress. This also suggests the quality of generated results may get worse when handling the larger datasets. It is worth noting that all generative models with satisfactory effects have a parameter pair within ratio $\frac{b}{2}\leq a\leq b$ as we suggest in Section 3.3. Another advice we give in the analysis of parameter selection is a,b≤1, and there exist some models without such constraint which can still converge. However, the loss curves of these models look not good as shown in Figure 3.

**Figure 4:**
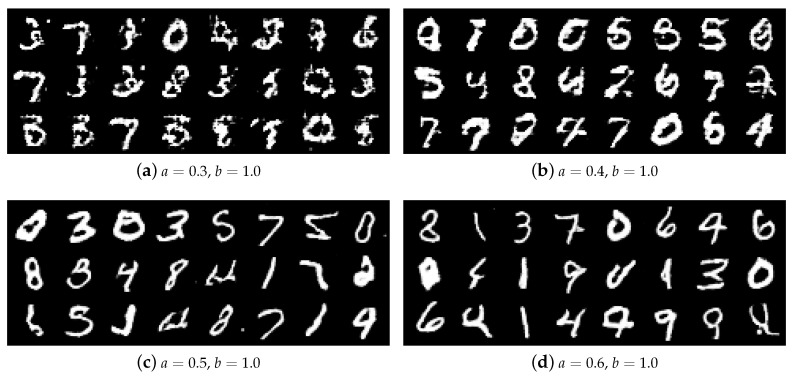
Generated samples on MNIST dataset with different parameters settings. a,b denote the two hyper-parameters in the objective function.

To show the effect of hyper-parameters on Alpha-GAN more clearly, some of the generated results are illustrated in Figure 4. The index *a* is gradually set as 0.3,0.4,0.5 and 0.6 while *b* is fixed at 1. One intuitive phenomenon is that the quality of generated samples is becoming better when we increase the value of *a*. The image samples are very fuzzy and difficult to distinguish when a=0.3, and a=0.4 performs better. When a=0.5,0.6, the model can generate high-quality handwritten digits. As we interpreted before, a,b represent the restraint level of $D(x_{\mathrm{real}})$ and $D(x_{\mathrm{fake}})$ respectively in Alpha-GAN. Decreasing the value of *a* means reducing the gradient feedback of critic output on real data. In that case, the discriminator will learn less information from the ground-truth data, the generated results will lack diversity and become unreal.

One subsequent question arises naturally that "Can the value of *a* be arbitrarily large to generate decent and recognizable samples?". In our experiments, we explore another property of Alpha-GAN, which indicates that the loss curve becomes less stable when *a* or *b* increases. Especially when one of the parameters is bigger than 1, the output loss becomes extremely large and model is less possible to converge. For example, the final output loss of discriminator is beyond 1e10 in Figure 3c. Therefore, it is essential to strike a balance between the training stability and the desired quality of generated images.

### 4.5. Generation Results

We further show the generated results on the real-world datasets SVHN and CelebA, and compare our Alpha-GAN model with some baseline approaches, including WGAN, and WGAN-GP. Models all run in the same network architecture with fine-tuning.

#### 4.5.1. Comparison with Baseline Models

In this section, we conduct extensive experiments to compare our Alpha-GAN with some baseline generative models. The Fréchet Inception Distance is calculated for each generator trained on the CelebA dataset. As aforementioned, lower FID score means GANs can generate samples closer to real data. We randomly sample 10,000 images with each GAN model and calculate the corresponding FID scores on ground-truth dataset with over 200,000 images.

**Figure 5:**
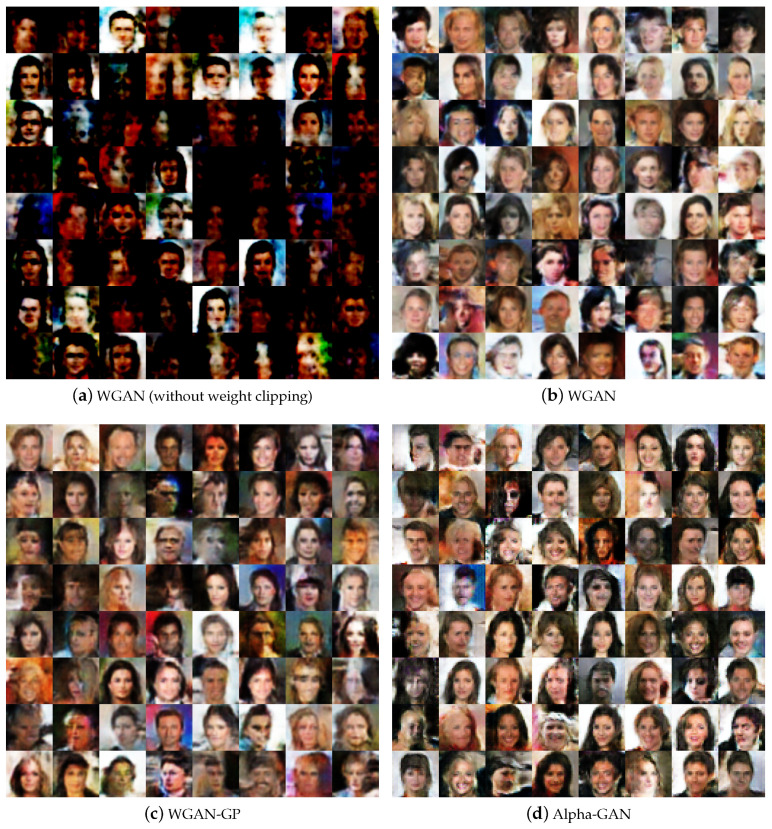
Generated samples of different models on CelebA dataset.

Figure 5 shows the generated results on CelebA dataset of our model and some competitors. Figure 5a illustrates samples generated by WGAN without weight clipping method and the results are not quite good. According to the theoretical analysis of WGAN, the weight clipping ensures the 1-Lipschitz continuous property of discriminator and convergence stability. This explains the poor quality of shown images. The samples of original WGAN is shown in Figure 5b, we observe that the results become better but still not good enough. In Figure 5c, the results of WGAN-GP have higher quality and are clear to be recognized. Similarly, our Alpha-GAN could generate competitive samples without any gradient penalty applied. Table 3 shows the Fréchet Inception Distance of our Alpha-GAN and some prominent GAN models on CelebA and SVHN dataset. Our proposed Alpha-GAN clearly outperforms WGAN and WGAN-GP.

#### 4.5.2. Generated Results

**Figure 6:**
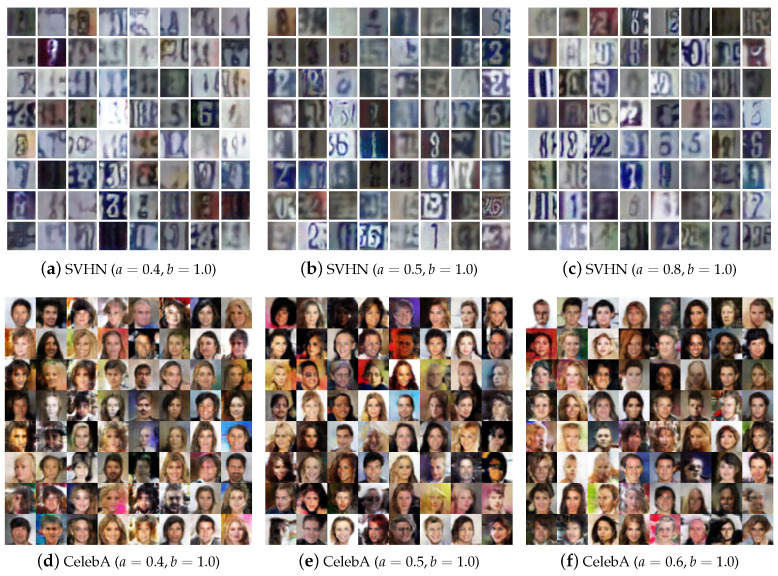
Generated samples of AlphaGAN.

We also evaluate our model on SVHN and CelebA with several different hyperparameter settings. The generated samples are shown in Figure 6. As can be seen, Figure 6a–c illustrate some sample images with a=0.4, a=0.5 and a=0.8 respectively. As the value of *a* increases, the digits figures become clear and recognizable. Figure 6d–f show some generated results on CelebA with a=0.4, a=0.5 and a=0.6 respectively. When the dataset becomes more complex and the network architectures go deeper, the increasing value of *a* can bring more instability on the results as we stated before.

## 5. Conclusions

In this paper, we propose a novel value function for GAN framework using the alpha divergence which can be regarded as a generalization of the Kullback–Leibler divergence. To improve Wasserstein-GAN, our objective introduces two more hyper-parameters to keep a balance during the training procedure. Moreover, we conduct a theoretical analysis for selecting appropriate hyper-parameters in order to control the information of $p_{\mathrm{data}}$ and $p_{g}$ to maintain the training stability. Furthermore, we also find some trade-off between the training convergence and generation quality. Experimental results demonstrate that attempts to generate extremely high-quality images may bring instability to GANs. A novel mechanism for explicitly controlling the two properties is explored and outperforms previous works. For future works, we hope to extend Alpha-GAN to large-scale datasets such as CIFAR10 and ImageNet.

## Figures and Tables

**Table 1:** Special cases in Amari-α divergence family.

α	Form	Divergence
α→−1	$\frac{1}{2}\int \frac{{\left(q\left(\theta \right)-p\left(\theta \right)\right)}^{2}}{p(\theta )}d\theta$	Reverse $\chi^{2}$ divergence
α→0	$\int q\left(\theta \right)\log\left(\frac{q(\theta )}{p(\theta )}\right)d\theta$	Kullback–Leibler divergence
α→1	$\int p\left(\theta \right)\log\left(\frac{p(\theta )}{q(\theta )}\right)d\theta$	Reverse KL divergence
$\alpha \to \frac{1}{2}$	$2\int {\left(\sqrt{p(\theta )}-\sqrt{q(\theta )}\right)}^{2}d\theta$	Hellinger divergence
α→2	$\frac{1}{2}\int \frac{{\left(p\left(\theta \right)-q\left(\theta \right)\right)}^{2}}{q(\theta )}d\theta$	Pearson $\chi^{2}$ divergence

**Table 2:** Convergence ability on different value selections of parameters.

Parameters	b=0.2	b=0.4	b=0.6	b=0.8	b=1.0	b=1.5	b=2.0
a=0.2	×	×	×	×	×	×	×
a=0.4	×	*√*	*√*	*√*	–	–	×
a=0.6	×	–	*√*	*√*	*√*	×	×
a=0.8	×	×	–	*√*	*√*	*√*	×
a=1.0	×	×	×	–	*√*	*√*	×
a=1.5	×	×	×	×	×	*√*	*√*
a=2.0	×	×	×	×	×	×	*√*

**Table 3:** Frechet Inception Distance of various models on CelebA and SVHN.

Model	CelebA	SVHN
WGAN [12]	181.11	37.02
WGAN-GP [13]	193.68	50.7
Alpha-GAN (ours)	176.37	23.26
